# Thymoma (World Health Organization Type AB) in multiple endocrine neoplasia type 1: a case report

**DOI:** 10.1093/jscr/rjac290

**Published:** 2022-07-08

**Authors:** Zheyuan Fan, Ling Wang, Jin Wang, Chundong Gu

**Affiliations:** Department of Thoracic Surgery, The First Affiliated Hospital of Dalian Medical University, Dalian, Liaoning, China; Department of Cardiothoracic Surgery, The Second People's Hospital of Guiyang, Guiyang, Guizhou, China; Department of Emergency Medicine, The First Affiliated Hospital of Dalian Medical University, Dalian, Liaoning, China; Department of Thoracic Surgery, The First Affiliated Hospital of Dalian Medical University, Dalian, Liaoning, China; Lung Cancer Diagnosis and Treatment Center of Dalian, The First Affiliated Hospital of Dalian Medical University, Dalian, Liaoning, China; Department of Thoracic Surgery, The First Affiliated Hospital of Dalian Medical University, Dalian, Liaoning, China; Lung Cancer Diagnosis and Treatment Center of Dalian, The First Affiliated Hospital of Dalian Medical University, Dalian, Liaoning, China

## Abstract

Thymic neoplasms are rarely seen among patients with multiple endocrine neoplasia type 1 (MEN1) and appear to be especially rare when pathological examination reveals a World Health Organization Type AB thymoma. In the case presented here, we report a 39-year-old woman with Type AB thymoma in MEN1. A 7.8-cm-sized mediastinal mass was diagnosed as a thymic neoplasm by computed tomography. In addition, pituitary tumor and hypercalcemia from parathyroid hyperplasia were found. Therefore, the patient was clinically diagnosed with MEN1 syndrome and underwent surgical resection of thymic tumor. At the 1-year follow-up, the patient appeared to be healthy without any sign of reoccurrence. Despite its rare occurrence, our case provides us with a new awareness that thymoma may coexist with MEN1.

## INTRODUCTION

Multiple endocrine neoplasia type 1 (MEN1) syndrome is an autosomal dominant genetic disorder characterized by primary hyperparathyroidism (PHPT), pituitary tumors and pancreatic neuroendocrine tumors. The prevalence of the disorder has been estimated at ~1–3 per 100 000. The tumor suppressor gene, MEN1, located within chromosome *11q13*, consists of 10 exons that encode a 610-amino acid protein named Menin, whose main functions have important roles in transcriptional regulation, genomic stability, cell division and cell cycle control [1–3]. MEN1 syndrome can initially manifest clinically as PHPT, pituitary tumors or pancreatic neuroendocrine tumors with nearly 100% penetrance by the age of 50 years [4]. Other less typical manifestations of MEN1 include lipomas, adrenal and thyroid tumors, cutaneous angiofibromas and bronchial or thymic tumors [1]. Furthermore, the majority of thymic epithelial tumors are thymic carcinoids, and thymoma patients with MEN1 are rare [5]. We describe a case involving a woman with MEN1 and thymoma (World Health Organization (WHO) Type AB) in this report.

## CASE REPORT

**Figure 1:**
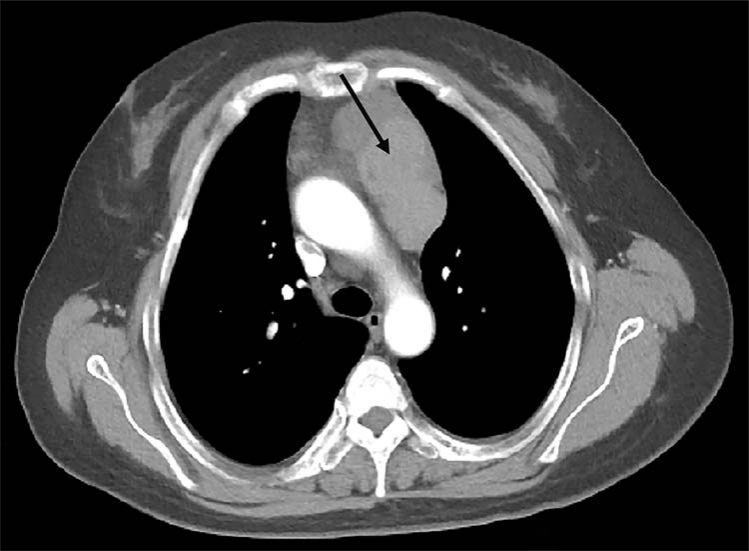
Enhanced CT scan of the chest revealed an anterior mediastinal tumor (black arrow).

**Figure 2:**
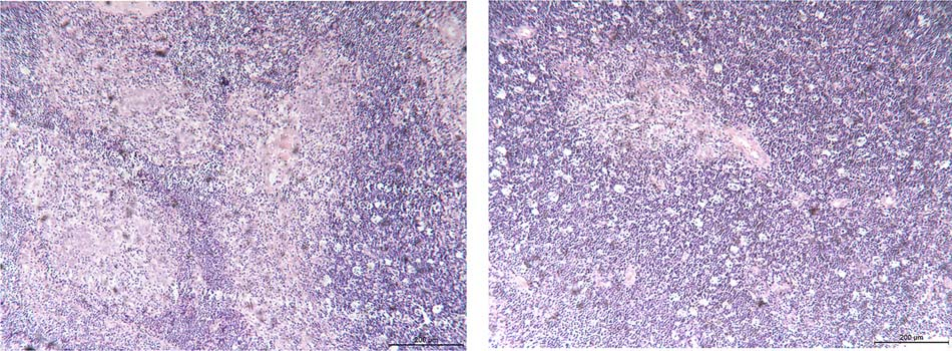
Histological findings of the surgical specimens; thymoma; (hematoxylin–eosin stain; original magnification ×100).

A 39-year-old Chinese female was diagnosed with a pituitary tumor at age 18 when she noticed aberrant growth development. Throughout the years, she developed PHPT, gastrinoma and bilateral adrenal hyperplasia. She was referred to our hospital for further evaluation to confirm the diagnosis of MEN1. Enhanced computed tomography (CT) demonstrated a 7.8-cm by 3.5-cm irregular anterior mediastinal solid-cystic mass within the thymic region (Fig. 1). The lesion was highly suspicious for thymic neoplasm radiologically. The patient subsequently underwent a video-assisted thoracoscopic thymectomy under general anesthesia. No tumor invasion to the pericardium and lung was observed, and the tumor was completely removed. Histopathology examination revealed WHO Type AB thymoma (Masaoka Stage I) (Fig. 2). Following surgery, annual screening showed no evidence of local recurrence or distant metastasis.

## DISCUSSION

We present an extremely rare case of a patient with WHO Type AB thymoma with MEN1. To the best of our knowledge, this finding is the first ever reported. Most of the neoplasms of the thymus gland which have been reported in MEN1 are thymic carcinomas. Only six cases of thymoma in MEN1 have been reported in the literature. Akua Graf *et al*. reported a case of an aggressive thymoma (Type B3) in a patient with MEN1 syndrome [6]. Tomita *et al*. reported a Type B3 thymoma with neuroendocrine differentiation in MEN1, and there were four previous cases in the literature which describe thymoma in MEN1 [5].

Clinically, the presence of thymoma in some MEN1 patients may go unreported until found on abnormal chest CT imaging. To date, the explanation of this rare combination of thymoma and MEN1 remains unclear.

In summary, regular and thorough examinations of MEN1 patients are required and should be carried out to detect thymic tumors early because they can be a substantial cause of mortality. Further studies to elucidate the pathogenesis of thymic epithelial tumors in MEN1 and their clinical significance remain to be performed.

## AUTHORS’ CONTRIBUTIONS

Conception was by Z.F., and C.G.; data acquisition and analysis were done by Z.F., L.W. and J.W.; writing the article was done by Z.F. and L.W.; All authors read and approved the final article.
